# Association of triglyceride glucose index with sepsis risk after major abdominal surgery: A retrospective cohort study

**DOI:** 10.12669/pjms.41.6.12187

**Published:** 2025-06

**Authors:** Qin Xia, Ye Wang, Di Wu, Zhou Lv

**Affiliations:** 1 Qin Xia Department of Anesthesiology and Surgical Intensive Care Unit, Xinhua Hospital Affiliated to Shanghai Jiao Tong University School of Medicine, Shanghai 200092, P.R. China; 2 Ye Wang Clinical Medical School, Xinhua Hospital Affiliated to Shanghai Jiao Tong University School of Medicine, Shanghai 200092, P.R. China; 3 Di Wu Department of Anesthesiology and Surgical Intensive Care Unit, Xinhua Hospital Affiliated to Shanghai Jiao Tong University School of Medicine, Shanghai 200092, P.R. China; 4 Zhou Lv Department of Anesthesiology and Surgical Intensive Care Unit, Clinical Research Unit, Xinhua Hospital Affiliated to Shanghai Jiao Tong University School of Medicine, Shanghai 200092, P.R. China

**Keywords:** Abdominal surgery, Insulin resistance, Sepsis, Triglyceride-glucose index

## Abstract

**Background & Objective:**

Recent studies have showed a correlation between hyperglycemia and insulin resistance with adverse outcomes in multiple critical diseases, including sepsis. The triglyceride-glucose index (TyGi) is now recognized as a proxy indicator of insulin therapy resistance. We aimed to ascertain the connection between TyGi and the sepsis prevalence and clinical outcomes in patient’s post-abdominal surgery.

**Method:**

Data for this retrospective cohort study was acquired from the Medical Information Mart for Intensive Care IV database from 2008 to 2019. Patients (≥18 years) who had elective major abdominal surgery were included. The primary outcome was the occurrence of sepsis following abdominal surgery. The connection between TyGi and sepsis incidence was investigated with multivariable Cox regression analysis.

**Results:**

One thousand eight hundred eighty-four patients were included in this study. The cumulative incidence of sepsis during hospitalization was 12.3%. The adjusted Cox regression model showed that raised TyGi levels were linked to a greater probability of sepsis incidence (Hazard’s ratio, 1.907; 95% CI, 1.327-2.739; p<0.001). Restricted Cubic Spline analysis demonstrated that TyGi possessed a strong and almost linear connection with the likelihood of postoperative sepsis. Subgroup analysis showed interaction effects in the subgroup with low high-density lipoprotein cholesterol (p for interaction=0.018). Furthermore, the incorporation of TyGi into the existing prediction model shows an enhancement in outcome prediction. The C-statistic elevated from 0.696 to 0.722, p<0.007. The continuous net reclassification improvement (NRI) was 0.203, p=0.005, and the integrated discrimination improvement (IDI) was 0.007, p<0.001.

**Conclusion:**

Patients at increased risk of developing sepsis following abdominal surgery may be identified in clinical practice with TyGi.

## INTRODUCTION

Sepsis is a deregulated inflammatory and immune response to microbial infection that is associated with high mortality rates, particularly in high-risk populations, such as elderly and post-surgical patients.^1^ Pathological changes caused by sepsis incorporate elevated levels of insulin-antagonistic hormones, excessive release of inflammatory mediators and lipid-derived factors, and oxidative and endoplasmic reticulum stress, which together lead to stress hyperglycemia and insulin resistance (IR).^2^ Studies show that IR and secondary hyperglycemia in sepsis are adaptive responses to sepsis-induced hypo-perfusion and reduced blood flow that provides the body with glucose necessary for tissue repair and inflammatory response.^3^

However, a growing body of evidence shows the correlation between hyperglycemia and IR with adverse outcomes in critically ill patients. An increased susceptibility to infections and sepsis is found in individuals diagnosed with diabetes mellitus,^4^,^5^ accounting for 20.1-22.7% of all sepsis cases.^6^,^7^ Thus, the possible significance of IR as a risk factor for developing sepsis in severely sick individuals justifies more investigation.

The triglyceride-glucose index (TyGi) is a straightforward and precise proxy quantification of IR computed as the logarithmized product of fasting triglycerides (TG) and fasting glucose. Prior investigations demonstrated that the TyGi may serve as a potential indicator of adverse clinical outcomes in cases of cardiovascular diseases, atherosclerosis, and certain metabolic disorders.^8^-^12^ This retrospective cohort investigation aimed to estimate the connection of TyGi with the sepsis incidence after abdominal surgery and to estimate the possible connection between IR and the sepsis risk in this patient group.

## METHODS

The present retrospective cohort research implemented data obtained from the Medical Information Mart for Intensive Care IV (MIMIC-IV), an online public database. The MIMIC-IV dataset provides accurate and superior data on the clinical characteristics of patients in the ICUs at Beth Israel Deaconess Medical Center from 2008 to 2019.

### Ethical Statement:

Authorization to access the database was given to the author ZL with certification number 42669087 (Date: May 24, 2021).

This study was conducted at Xinhua Hospital Affiliated to Shanghai Jiao Tong University School of Medicine from December 2023 to May 2024, followed the reporting instructions stated in the Strengthening the Reporting of Observational Studies in Epidemiology (STROBE) guidelines.

### Inclusion and exclusion criteria:

Eligibility was limited to adult patients who were seeking elective major abdominal surgery. Upper gastrointestinal, hepato-biliary-pancreatic, colorectal, urinary, and gynecological surgeries and exploratory laparotomy were among the procedures performed. Procedures were classified according to the International Classification of Diseases 9th and 10th Edition (ICD-9 and ICD-10) codes. Additional file Table A1. All patients received standardized perioperative antimicrobial treatment and were investigated with blood culture to determine infection status. Patients who did not have available TG and glucose values were eliminated. For a patient having multiple hospitalizations due to different abdominal surgical procedures, only the first surgery exposure was analyzed.

### Data extraction:

Baseline characteristics within 24 hours after hospital admission were collected, which included demographics (age, gender, smoking status, abdominal obesity), comorbidities [hypertension, diabetes, chronic kidney disease (CKD), coronary artery disease (CAD), congestive heart failure (CHF), atrial fibrillation (AFIB), chronic obstructive pulmonary disease (COPD), malignancy, hepatic disease, and Charlson comorbidity index (CCI)], and laboratory variables [TG, fasting blood glucose (FBG), high-density lipoprotein cholesterol (HDL-C), albumin, white blood cell (WBC) count, platelet counts, hemoglobin, creatinine and total bilirubin (TB)]. If a given factor was reported several times during the first 24-hour period after admission, the value indicating the greatest severity was chosen. Additional data collected included the duration of hospitalization, postoperative admission to the intensive care unit (ICU), and the need for invasive breathing and vasopressors.

### Outcomes and covariates:

The primary independent factor was the TyGi at admission; the TyGi was determined by using the formula: TyGi =ln [Fasting triglyceride (mg/dl) × fasting glucose (mg/dl)]/2.^13^ The reference group was the smallest quartile of the TyGi, which was employed as both a continuous and categorical variables. Covariates that were adjusted for in the regression models included demographic characteristics, comorbidities, and laboratory variables. The primary outcome was the cumulative incidence of sepsis after surgical procedures during hospitalization. Sepsis-3 criteria were used to define sepsis patients, which included documented or suspected infection and an increase in Sequential Organ Failure Assessment (SOFA) by ≥ 2 points.^14^ Infection was identified using the ICD-10 codes in MIMIC-IV. Additional file Table A2. Secondary outcomes included in-hospital mortality in the overall population and the sepsis subset, ICU admission, and the requirement for invasive ventilation and vasopressors.

In this retrospective analysis, values of continuous variables that were considered implausible or outlying (below 5%) were substituted with average or median values. Missing data, accounting for below 30% of the remaining variables, were analyzed with multiple imputations. In the case of categorical variables with incomplete information, dummy variables were established. The independent-sample t- or Mann-Whitney U-tests were implemented to express continuous data, which were stated as means with standard deviations or medians followed by interquartile ranges, respectively. Expressed as percentages, values representing categorical variables were ascertained with either the chi-square or Fisher’s exact tests. Statistical analysis of Kaplan-Meier survival was conducted, with the log-rank test to investigate variations across various TyGi levels.

A multivariable Cox regression analysis was performed to assess the correlation between the TyGi and clinical outcomes. The multivariable regression models included the baseline factors that were deemed significant from a clinical standpoint. Multicollinearity was ascertained with the variable inflation factor (VIF) approach, where a VIF value more than or equal to five indicates multicollinearity. Three multivariable regression models were created to establish connections between the TyGi and prognoses: Model 1: unadjusted; Model 2: demographically adjusted for age, gender, current smoking status, abdominal obesity, comorbidities, and CCI; Model 3: laboratory variables adjusted based on model 2. An analysis was conducted with restricted cubic splines (RCS) with four knots to estimate any connections between the TyGi and the sepsis occurrence, as well as the mortality rate in hospital.

Investigation of the association between TyGi and prognoses of major abdominal surgery patients, as well as metabolic disorders, was performed with subgroup analysis. Stratification was determined by metabolic state upon hospital admission, taking into account blood pressure equal to or less than 130/85mmHg, FBG equal to or below 100mg/dL, plasma TG equal to or below 150mg/dL, and serum HDL-C levels equal to or less than 40mg/dL for men, and equal to or less than 50mg/dL for females. In the sensitivity analysis, adjustments were performed for the factors in model-3 as well as the history of lipid-lowering or hypoglycemic drugs. We quantitatively assessed the predictive efficacy of the TyGi in predicting sepsis incidence by calculating the concordance index (C-index), continuous net reclassification improvement (NRI), and integrated discrimination improvement (IDI) for two models: Model 3 with the TyGi and Model 3 without it.

### Statistical analysis:

Graphical representation and statistical analysis were conducted with SPSS (IBM SPSS 26.0, SPSS Inc) and R software version 4.2.2 (R Foundation for Statistical Computing, Auckland, New Zealand). The graphic abstract was created using the Fig draw version 2.0 online program.

## RESULTS

We examined a cohort of 1884 individuals who had significant abdominal operations. Additional file Fig.A1. In Table-I, the baseline characteristics for both non-septic and septic individuals are shown. Advanced age and a greater prevalence of diabetes, COPD, CHF, CKD, CAD, AFIB, and hepatic disease were found in septic patients. In terms of metabolic syndrome (MetS) indicators, septic patients exhibited higher levels of TG, higher rates of abdominal obesity, lower HDL-C levels, and lower FBG levels than non-septic patients.

**Fig.1:**
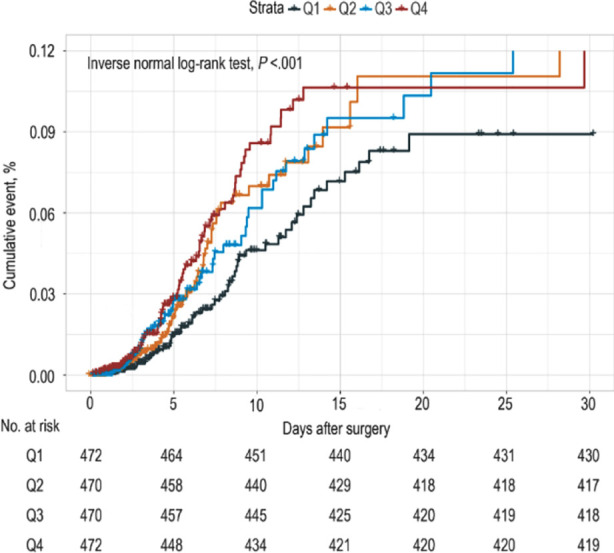
The cumulative event incidence curves for incidence of sepsis. TyG index quartile Q1: ≤8.46; Q2: 8.47–8.83; Q3: 8.84–9.21; Q4: ≥9.22

**Table-I:** The baseline characteristics and outcomes for patients with and without sepsis after abdominal surgeries.

Variables	All patients (n=1884)	Non-sepsis (n=1653)	Sepsis (n=231)	P value
Age	59(48-69)	59(48-69)	63(49-75)	0.002
Gender(male), n(%)	991(52.6)	848(51.3)	143(61.9)	0.002
Current smoker, n(%)	153(8.1)	126(7.6)	27(11.7)	0.034
Abdominal obesity, n(%)	199(10.6)	106(6.4)	93(40.3)	<0.001
***Comorbidities***				
Hypertension	841(44.6)	734(44.4)	107(46.3)	0.583
Diabetes	404(21.4)	342(20.7)	62(26.8)	0.033
COPD	35(1.9)	26(1.6)	9(3.9)	0.005
CHF	113(6.0)	77(4.7)	36(15.6)	<0.001
CKD	117(6.2)	93(5.6)	24(10.4)	0.005
CAD	211(11.2)	167(10.1)	44(19.0)	<0.001
AFIB	205(10.9)	147(8.9)	58(25.1)	<0.001
Hepatic disease	116(6.2)	92(5.6)	24(10.4)	<0.001
Malignancy	827(43.9)	736(44.5)	91(39.4)	0.141
CCI	4(2-6)	4(2-6)	6(3-8)	<0.001
***Laboratory tests***				
HDL-C (mg/dL)	45(36-57)	46(37-57)	38(28-50)	<0.001
TG (mg/dL)	117(86-170)	116(86-167)	134(93-196)	<0.001
Fasting glucose(mg/dL)	84(76-92)	85(77-93)	81(71-87)	<0.001
TyG index	8.83(8.50-9.21)	8.82(8.46-9.19)	8.90(8.48-9.36)	0.043
Albumin (g/dL)	3.0(2.3-4.0)	3.4(2.5-4.1)	2.2(1.9-2.7)	<0.001
WBC(K/uL)	9.5(8.0-12.2)	9.1(7.9-11.8)	12.9(11.2-16.6)	<0.001
Hemoglobin (g/dL)	10.4(8.4-12.0)	10.7(8.7-12.2)	8.0(7.3-9.0)	<0.001
Platelet (K/uL)	149(92-200)	155(102-205)	94(45-151)	<0.001
Creatinine(μmol/L)	1.2(1.0-2.1)	1.2(0.9-1.8)	2.1(1.3-4.2)	<0.001
Bilirubin(μmol/L)	0.8(0.5-2.0)	0.8(0.5-1.5)	2.5(0.9-8.6)	<0.001
***Type of surgery***				
Upper gastrointestinal, n(%)	346(18.4)	323(19.5)	23(10.0)	<0.001
Hepato-biliary-pancreatic, n(%)	256(13.6)	212(12.8)	44(19.0)	0.010
Colorectal surgery, n(%)	665(35.3)	615(37.2)	50(21.6)	<0.001
Urinary surgery, n(%)	242(12.8)	233(14.1)	9(3.9)	<0.001
Gynecological surgery, n(%)	19(1.0)	19(1.1)	0(0)	0.082
Exploratory laparotomy, n(%)	356(18.9)	251(15.2)	105(45.5)	<0.001
Laparoscopic, n(%)	603(32.0)	591(35.8)	12(5.2)	<0.001
***Outcomes***				
Death in hospital, n(%)	84(4.5)	32(1.9)	52(22.5)	<0.001
ICU admission, n(%)	421(22.3)	210(12.7)	211(91.3)	<0.001
Invasive ventilation, n(%)	238(12.6)	90(5.4)	148(64.1)	<0.001
Vasopressor use, n(%)	243(12.9)	105(6.4)	138(59.7)	<0.001
LOS, (days)	6.2(3.3-13.2)	5.4(3.2-10.1)	21.4(12.2-34.2)	<0.001

COPD chronic obstructive pulmonary disease; CHF congestive heart failure; CKD chronic kidney disease; CAD coronary artery disease; AFIB atrial fibrillation; CCI Charlson Comorbidity Index; HDL-C high-density lipoprotein cholesterol; TG triglyceride; TyG index triglyceride-glucose index; WBC white blood cell; ICU intensive care units; LOS length of stay

Notably, septic patients showed a significantly higher TyGi value as opposed to non-septic patients [8.90 (8.48-9.36) vs 8.82 (8.46-9.19); p=0.043]. Postoperative sepsis was more common in patients with hepato-biliary-pancreatic surgery (12.8% vs. 19.0%; p=0.010) and exploratory laparotomy (35.8% vs. 5.2%; p<0.001) than patients undergoing other surgical procedures. In addition, patients with postoperative sepsis had a significantly greater in-hospital mortality rate (22.5% vs. 1.9%; p<0.001), a greater admission rate to the ICU (91.3% vs. 12.7%; p<0.001), and a greater distribution of invasive ventilation (64.1% vs 5.4%; p<0.001) and vasopressors (59.7% vs 6.4%; p<0.001). Significantly longer hospital stays were related to sepsis (21.4 vs 5.4; p<0.001). Table-I

### Primary outcome:

A cohort of 231 patients, accounting for 12.3%, had postoperative sepsis. Cox regression analysis revealed a positive correlation between a one-unit rise in the TyGi and an elevated likelihood of postoperative sepsis [Hazards ratio (HR), 1.334; 95%CI, 1.069-1.665; p=0.011]. After adjusting demographic characteristics, comorbidities, and laboratory variables, TyGi remains an independent risk factor for sepsis incidence following abdominal surgery (HR, 1.907; 95%CI, 1.327-2.739; p<0.001) (Table-II).

**Table-II:** The relationship between TyG index and incidence of sepsis.

Categories	Model 1			Model 2			Model 3		
	HR (95%CI)	P	P for trend	HR (95%CI)	P	P for trend	HR (95%CI)	P	P for trend
Per 1 Unit increase	1.334 (1.069-1.665)	0.011		1.496 (1.170-1.913)	0.001		1.907 (1.327-2.739)	<0.001	
Quartile^a^			0.074			0.014			0.001
Q1(n=472)	Ref			Ref			Ref		
Q2(n=470)	1.005 (0.671-1.505)	0.981		1.340 (0.841-2.135)	0.077		1.708 (0.941-3.103)	0.079	
Q3(n=470)	1.006 (0.677-1.512)	0.983		1.379 (0.861-2.207)	0.181		1.889 (1.014-3.521)	0.045	
Q4(n=472)	1.423 (0.973-2.081)	0.069		1.785 (1.140-2.796)	0.011		2.969 (1.576-5.595)	0.001	

Model 1: unadjusted; Model 2: adjusted for age, gender, current smoking, abdominal obesity, comorbidities (Hypertension, Diabetes, COPD, CKD, CHF, CAD, AFIB, Hepatic disease and Malignancy), CCI; Model 3: adjusted for age, gender, current smoking, abdominal obesity, comorbidities (Hypertension, Diabetes, COPD, CKD, CHF, CAD, AFIB, Hepatic disease and Malignancy), CCI, HDL-C, albumin, hemoglobin, platelet, WBC, creatinine, bilirubin;

a TyG index quartile Q1: ≤8.46; Q2: 8.47–8.83; Q3: 8.84–9.21; Q4: ≥9.22

According to their TyGi, patients were categorized into four groups: Q1 (TyGi ≤8.46), Q2 (8.46 TyGi ≤8.83), Q3 (8.83 TyGi ≤9.21), and Q4 (TyGi > 9.21). Compared to the TyGi value in Q1 (Table-II, p for trend = 0.074), there was no significant correlation observed between the occurrence of sepsis in any of the groups. After adjusting for confounding factors, a significant trend of increasing risk of sepsis in quantiles of the TyGi was presented in model 2 (p for trend = 0.014) and model 3 (p for trend = 0.001). Table-II Moreover, Kaplan–Meier analysis curves suggested that the sepsis cumulative incidence possessed a significant variation across the groups throughout the follow-up interval (Fig.1).

### Detection of connection between TyGi and the incidence of sepsis:

**Fig.2:**
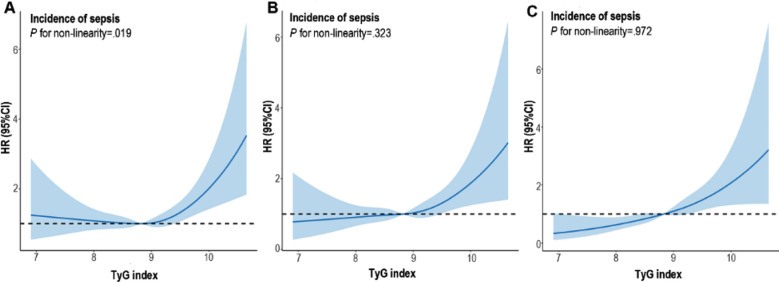
Restricted cubic spline regression for the TyG index and sepsis incidence. A: Unadjusted restricted cubic spline (RCS) model for sepsis incidence. B: Adjusted RCS model for sepsis incidence [adjusted for age, gender, current smoking, abdominal obesity, comorbidities (hypertension, diabetes, COPD, CKD, CHF, CAD, AFIB, hepatic disease, and malignancy), CCI]. C: Adjusted RCS model for sepsis incidence (adjusted for age, gender, current smoking, abdominal obesity, comorbidities (hypertension, Diabetes, COPD, CKD, CHF, CAD, AFIB, hepatic disease, and malignancy), CCI, HDL-C, albumin, hemoglobin, platelet, WBC, creatinine, bilirubin). HR, odds ratio; CI, confidence interval; TyG, triglyceride-glucose

The unadjusted RCS model implicated a U-shaped link between TyGi and postoperative sepsis. Fig.2A. After excluding possible confounding influences of demographic characteristics, comorbidities, and laboratory variables, the curve tended to be linear. Fig.2B and 2C.

### Secondary Outcomes:

**Fig.3:**
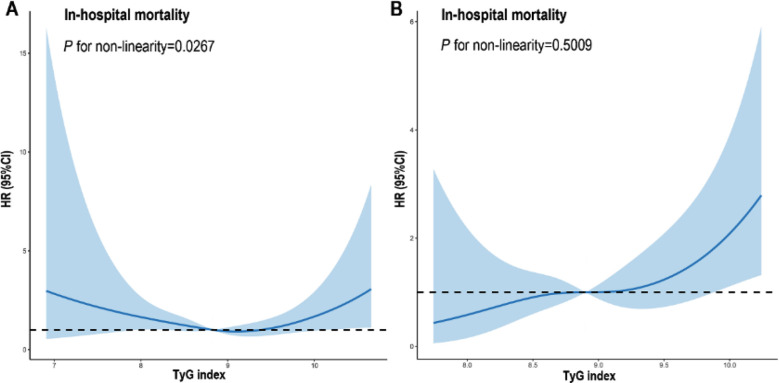
Restricted cubic spline for the in-hospital mortality. A: Restricted cubic spline for the in-hospital mortality of the whole study population; B: Restricted cubic spline for the in-hospital mortality of the septic patients.

During hospitalization, a total of 84 participants (4.5%) died. RCS regression analysis showed TyGi possessed an approximately U-shaped connection with in-hospital mortality in the overall study population. Fig.3A. Significantly, the RCS model showed that the condition of linearity regarding the link between TyGi and in-hospital mortality was not disproven in the sepsis subgroup (p for non-linearity=0.501) (Fig.3B).

Multivariable Cox regression analysis suggested that an independent association was found between TyGi and increased chances of ICU admission (HR, 1.658; 95%CI, 1.106-2.486; p=0.014), receipt of invasive mechanical ventilation (HR, 1.725; 95%CI, 1.106-2.691; p=0.016) and vasopressors (HR, 1.618; 95%CI, 1.090-2.590; p=0.019). These results were further confirmed by model 2 and model- 3.

### Subgroup and sensitivity analyses:

**Fig.4:**
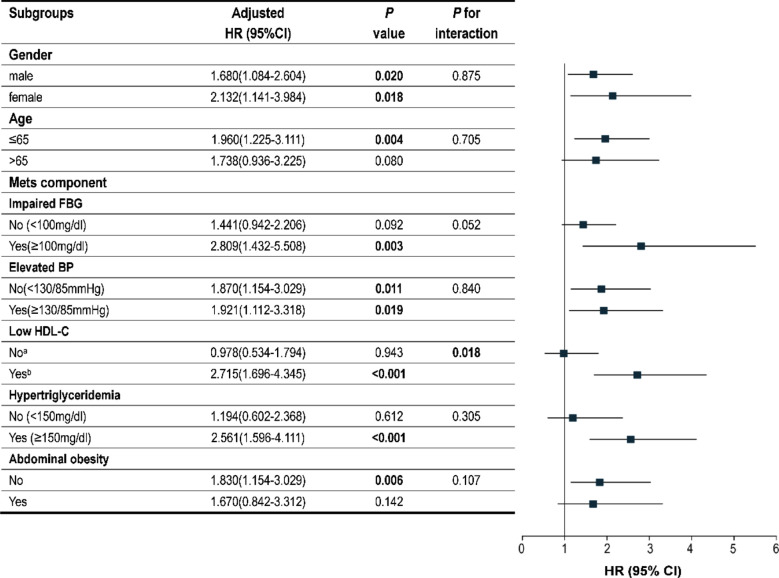
Subgroup analyses of adjusted HRs for sepsis after abdominal surgery. HR Odds ratio, CI confidence interval, Mets metabolic syndrome, FBG fasting blood glucose, BP blood pressure, HDL-C high-density lipoprotein cholesterol, aHDL-C (>=40mg/dl for male and >=50mg/dl for female), bHDL-C (< 40mg/dL for male and < 50mg/dL for female).

Subgroup analysis was performed using the gender, age, and metabolic conditions of individuals. Within these subgroups, the TyGi predictive value appeared more pronounced in patients with impaired FBG (HR range 1.432-5.508), low HDL-C levels (HR range 1.696-4.345), and hypertriglyceridemia (HR range 1.596-4.111), contrasted with the overall cohort. Significantly, the multivariate analysis revealed significant interactions between low HDL-C levels and TyGi in relation to the likelihood of sepsis occurrence (p for interaction=0.018). While there were no findings of interaction between the factors and TyGi in the other subgroups, the TyGi reported a significant link to a greater sepsis risk in subgroups categorized by gender (HR range 1.084-2.604 for males and 1.141-3.984 for females), age≤65 years (HR range 1.225-3.111), non-elevated blood pressure (HR range 1.154-3.029), elevated blood pressure (HR range 1.112-3.318), and absence of abdominal obesity (HR range 1.154-3.029).Fig.4 In addition, after adjusting for confounding variables in sensitivity analyses, the data exhibited steady behavior, therefore confirming the dependability of our conclusions.

### The predictive performance of the TyGi for sepsis incidence:

Introducing the TyGi into the fully adjusted model (Model-3) significantly improved the prediction accuracy of postoperative sepsis (C-index rose from 0.696 to 0.722; p<0.001). The continuous NRI study demonstrated a significant enhancement in classification [continuous NRI (95% CI):0.203 (0.096-0.379); p=0.005]. Moreover, the TyGi showed a significant improvement in accurately identifying those at risk of developing sepsis following major abdominal surgery [IDI (95% CI): 0.007 (0.003-0.011); p<0.001].

## DISCUSSION

In this study, we demonstrated a significant connection between the age and presence of comorbidities, including diabetes, COPD, CHF, CKD, CAD, AFIB, and liver disease, and the likelihood of sepsis development in patients who had abdominal surgery. One independent risk factor for postoperative sepsis and worse clinical outcomes was shown to be a high baseline TyGi. Integration of the TyGi into the existing risk prediction model, which already incorporated demographic data, comorbidities, and laboratory indicators, significantly improved its predictive accuracy. Compared with non-septic patients, patients with postoperative sepsis had significantly higher in-hospital mortality and ICU admission rates and a higher need for invasive ventilation and vasopressors, which is consistent with previous reports.^15^,^16^ Our results further add to the existing body of evidence identifying possible risk factors for sepsis.^17^,^18^ Diagnosis of diabetes in our study correlated with the risk of developing sepsis after the abdominal surgery. Our study confirms previous reports that show diabetes as a risk factor for sepsis. Diabetic patients are prone to developing sepsis due to their reduced immune cell function and predisposition to chronic ulcers, kidney disease, and diabetic angiopathy, which increases the risk of infections.^19^,^20^ However, as shown in the 2017 meta-analysis by Wang et al.^21^ that included 10 studies, the existence of diabetes did not impact the outcome of septic patients.

Interestingly, we show that elevated baseline levels of TG levels were more prevalent in patients who developed sepsis after the abdominal surgery. Previous studies have reported that pre-sepsis levels of TG were inversely linked to hospital mortality in septic patients.^22^,^23^ However, Maile et al.^24^ showed that too high or too low levels of TG both led to increased sepsis-associated mortality. Elevated baseline range of blood TG levels is a sensitive marker of IR,^25^ which is often accompanied by dyslipidemia and, therefore, may provide an indication of the potential risk of developing sepsis in patients after abdominal surgery. This potential association of pre-existing IR and a higher risk of post-surgery sepsis is further confirmed by our observation of the elevated baseline TyGi in septic patients. Previous investigations reported that patients may develop rapid-onset acute IR in minutes, hours, or days after the onset of sepsis.^26^

Moreover, the acute IR development in critically ill patients was related to the severity of their condition rather than to the accompanying comorbidities.^27^ However, the link between the pre-existing IR to the risk of sepsis in post-surgical patients is still unclear. Liao et al.^28^ included 3,026 patients and used TyGi as a marker of IR, showing that the connection between chronic IR and all-cause mortality in critically ill individuals was established. Another study by Liu et al.^29^ showed that the baseline serum TyGi was an independent risk factor for the initial episode of peritoneal dialysis-related peritonitis in chronic peritoneal dialysis patients. Upon controlling for confounding variables, our investigation demonstrated that baseline TyGi continued to be an independent risk factor for the occurrence of sepsis after abdominal surgery.

A baseline TyGi was significantly linked to an extended duration of ICU stay, the need for ventilator-assisted ventilation, and the use of vasopressors. Adjusted higher TyGi quantiles showed a linear association with the increased incidence of sepsis, and incorporating TyGi into the existing post-operative sepsis risk prediction model that included demographic characteristics, comorbidities, and laboratory variables led to a notable improvement in its prognostic performance. Furthermore, the predicted accuracy of the TyGi was greater in individuals with compromised baseline characteristics FBG, low HDL-C, and hypertriglyceridemia compared to the overall population in our study. Notably, multivariate analysis showed a significant correlation between low baseline HDL-C and the TyGi regarding the risk of sepsis after the surgery.

This correlation comes together with our observation that septic patients had significantly higher rates of MetS, manifested as elevated TG, higher rates of abdominal obesity, and lower HDL-C levels and FBG levels than non-septic patients. Numerous studies have showed the connection between MetS and sepsis and suggested that the existence of MetS may increase the risk of adverse outcomes, which can lead to a higher mortality rate in septic patients.^24^,^30^–^34^ Furthermore, recent research using single-cell data analysis and machine learning methods proposed that sepsis and MetS may have similar glucose metabolism-linked pathways.^35^ It is plausible, therefore, that the baseline TyGi as an indicator of IR may be used as a sensitive predictive marker of potential septic complications after the surgery.

We may propose a possible mechanism of the observed association between pre-existing IR, as indicated by elevated TyGi, and increased risk of sepsis after the abdominal surgery. TyGi reflects levels of endothelial dysfunction, oxidative stress, and inflammatory response.^36^-^38^ This is further confirmed by the studies that showed positive associations of the TyGi with WBC and high-sensitivity C-reactive protein levels.^10^,^39^ An already impaired immune function of the patients with pre-existing IR is further weakened by the abdominal surgery, making these patients more susceptible to infection. Subsequently, inflammation-impaired vascular endothelium cannot prevent the leakage of blood contents into the perivascular spaces, leading to further vascular damage (Pantoni). This theory is further confirmed by the observed correlation between the TyGi and the post-operative requirement of vasopressors in our study population.

The outcomes of our investigation may possess important clinical consequences. We show the direct correlation between the pre-existing IR, reflected by high TyGi, and the elevated sepsis risk in patients who undergo abdominal surgery. TyGi, therefore, can be included in the routine preoperative assessment of patients undergoing abdominal surgery to timely find patients at greater sepsis risk following the operation.

### Limitations:

Our work possesses several limitations. This was a retrospective investigation of data from the US patients, which impacts the robustness and generalizability of our results. Furthermore, we only focused on the predictive value of TyGi in cases of major abdominal surgery. Additional investigations are needed to investigate whether this connection remains significant for other major surgeries, such as cardiothoracic surgery or neurosurgical procedures.

## CONCLUSION

Patients who underwent abdominal surgery, a risk of developing postoperative sepsis correlated with comorbidities and significantly worse clinical outcomes. A high baseline TyGi has been shown to be an independent risk factor for postoperative sepsis and worse clinical outcomes. The incorporation of the TyGi significantly enhanced the existing risk prediction model. Hence, the TyGi may be used in clinical practice to detect patients who have a heightened susceptibility to sepsis after abdominal surgery.

### Authors’ contributions:

**QX:** Literature search, study design and manuscript writing.

**YW, DW and ZL:** Data collection, analysis and interpretation. Critical Review.

**ZL:** Was involved in the manuscript revision and validation and is responsible for the integrity of the study.

All authors have read and approved the final manuscript.
